# C-Reactive Protein Levels of Healthy Term Infants Born After Prolonged Rupture of Membranes

**DOI:** 10.3390/pediatric17060133

**Published:** 2025-12-05

**Authors:** Anders Batman Mjelle, Vilde Solberg, Emma Rød, Eydís Oddsdóttir Stenersen, Håvard Tetlie Garberg, Per Arne Tølløfsrud, Arild Rønnestad, Anne Lee Solevåg

**Affiliations:** 1 Department of Anaesthesia and Intensive Care, Haukeland University Hospital, 5021 Bergen, Norway; 2 Department of Quality and Health Technology, Faculty of Health Sciences, University of Stavanger, 4068 Stavanger, Norway; 3 Faculty of Medicine, University of Oslo, 0316 Oslo, Norway; 4 Department of Paediatric and Adolescent Medicine, Drammen Hospital, 3004 Drammen, Norway; 5 Department of Neonatal Intensive Care, Division of Paediatric and Adolescent Medicine, Oslo University Hospital, Rikshospitalet, 0424 Oslo, Norway; ptollofs@ous-hf.no (P.A.T.); a.e.ronnestad@medisin.uio.no (A.R.); 6 Institute of Clinical Medicine, Faculty of Medicine, University of Oslo, 0316 Oslo, Norway

**Keywords:** antibiotics, C-reactive protein, early-onset sepsis, neonate, prolonged rupture of membranes

## Abstract

Background/Objective: Even in the absence of infection, prolonged rupture of membranes (PROM) has been associated with elevated neonatal C-reactive protein (CRP). As both the sensitivity and specificity of CRP in predicting early-onset neonatal sepsis (EOS) may be low, we aimed to describe CRP levels during the first 36 h of life in term infants born after PROM ≥ 24 h. Methods: CRP was measured at 1, 12, and 36 h. Descriptive statistics and correlation analyses were performed, taking gestational age, birth weight, sex, delivery mode, and antibiotic treatment into account. Reference CRP values in healthy neonates without sepsis born after PROM were established. Results: Median (range) CRP was 0 (0–62) mg/L, 0 (0–82) mg/L, and 4 (0–92) mg/L at 1, 12, and 36 h, respectively. CRP at 12 and 36 (*p* < 0.001) but not 1 h was positively correlated with gestational age and birth weight. There was no difference in CRP after C-section vs. vaginal delivery. Among infants without sepsis, CRP was higher at all time points in infants who did vs. those who did not receive antibiotics (*p* < 0.001). Conclusions: CRP was low in term infants without sepsis born after PROM but with outliers above 60, 80, and 90 mg/L after 1, 12, and 36 h, respectively. Research is needed on the long-term outcomes of infants with inflammation, as evidenced by an elevated CRP after PROM.

## 1. Introduction

C-reactive protein (CRP) is a biochemical inflammation marker [1], and blood (serum or plasma) CRP is often used in early-onset neonatal sepsis (EOS) diagnostics [2]. Norwegian consensus guidelines define culture-negative sepsis as CRP > 30 mg/L combined with clinical signs of systemic infection and antibiotic treatment for ≥5 days [3].

However, elevated CRP may represent inflammatory conditions other than bacterial infection, and the sensitivity and specificity of CRP in predicting EOS may be low [2]. A Norwegian national survey indicated that only 5.9% of infants diagnosed with EOS, defined as within the first week of life, had positive blood cultures [4].

The limited association between moderately elevated CRP and proven invasive bacterial infections in the early neonatal period, as well as reports on elevated neonatal CRP in the absence of infection, underlines the difficulties in interpreting CRP in the first days of life [5,6]. Chiesa et al. reported that CRP (97.5th percentile) increased from 8.4 mg/L at birth to 26.5 mg/L at 48 h in healthy term or near-term neonates [7]. The time to peak value has been shown to be around 35 h for asymptomatic term infants [6]. A more recent study of healthy term infants showed that 7% had CRP ≥ 20 mg/L and 1.1% > 30 mg/L at 48–72 h of age [8]. Notably, several studies have shown that prolonged rupture of membranes (PROM) was associated with higher CRP values in the absence of infection [7,9], indicating an inflammatory response associated with PROM. As PROM is a risk factor for EOS, many hospitals have surveillance protocols to detect EOS in infants born after PROM and some recent research advocates for serial biochemical measurement, including of CRP [10]. However, it is worthwhile being aware that inflammatory markers, including CRP, may have a particularly limited specificity in EOS diagnostics in this population.

Interestingly, a large study based on the Medical Birth Registry and The Cerebral Palsy Register of Norway recently showed an association between PROM (≥24 h) and later development of cerebral palsy [11]. The authors proposed neonatal infection as the pathophysiological mechanism but had no information about infant diagnoses or antibiotic exposure. Thus, PROM might be an independent risk factor for cerebral palsy.

The aim of this study was to retrospectively examine CRP as a marker of inflammation in healthy term infants born after PROM and to assess the correlation between CRP, antibiotic exposure, and clinical characteristics. We hypothesized that elevated CRP is common after PROM, even in the absence of infection.

## 2. Materials and Methods

As part of a quality assurance initiative concerning different practices of EOS surveillance in four hospitals in two Norwegian healthcare trusts, CRP data were registered for term infants born after PROM [12].

The four hospitals, Rikshospitalet and Ullevål Hospital (Oslo University Hospital) and Bærum and Drammen Hospital (Vestre Viken Hospital Trust), cover a large fraction of the South-Eastern Norway Regional Health Authority population and were responsible for almost ¼ of deliveries in Norway during the study period [13]. Bærum Hospital does not have a neonatal intensive care unit (NICU). All infants with suspected EOS therefore needed to transfer to a hospital with a NICU, which was most commonly Drammen Hospital.

Rikshospitalet and Bærum Hospital used a serial measurement of CRP at 1–2, 12, and 36 h of life, in addition to the clinical observation of asymptomatic infants. The protocol did not specify specific CRP threshold values for suspecting EOS but left it at the attending pediatrician’s discretion. Ullevål and Drammen Hospital only measured CRP in the event of clinical symptoms and signs. Infants born in these two hospitals were therefore excluded from the analyses of CRP in the present study, but data were used for comparison regarding other variables and outcomes.

EOS was diagnosed by the growth of a pathogenic microbe in blood culture. In the absence of a positive blood culture, as per Norwegian consensus [3], the following diagnostic criteria had to be met: (1) clinical signs of infection, (2) CRP > 30 mg/L during the course of the disease, (3) ≥ 5 days of antibiotic treatment or death by clinical sepsis before 5 days, and (4) exclusion of alternative explanations for the infant’s symptoms.

### 2.1. Study Period, Participants, and Data Extraction

The study included all live-born infants with gestational age (GA) ≥ 37 + 0 weeks born after PROM ≥ 24 h in the four hospitals from 1 January 2017 to 31 December 2019. Exclusion criteria were prematurity or lack of PROM. No infant was excluded due to missing data.

Data were extracted from the electronic patient records DIPS (DIPS AS, Norway), Partus (CSAM, Oslo, Norway), and MetaVision electronic patient charts (iMDsoft, Tel Aviv, Israel). Extracted data included GA, birth weight (BW), sex, mode of delivery, Apgar scores, PROM duration, and antibiotic treatment.

Data were entered into IBM SPSS v29 (Armonk, NY, USA) and Microsoft Office Excel (Microsoft, Redmond, WA, USA). The data extractions were performed by three authors (ER, VS, EOS) between 2 January 2021 and 20 December 2021.

### 2.2. Ethical Considerations

The study was approved by the Data Protection Officer at Oslo University Hospital and Vestre Viken Hospital Trust and the Regional Committee for Medical and Health Research Ethics (reference number 148990: approved 28 September 2020. An extension of the project period was granted on 2 March 2023, 3 June 2024, and 2 July 2025. The project was approved until 31 July 2026). As the study was considered a quality assurance initiative, we were not required to collect individual consent from the parents of the included infants.

### 2.3. CRP Measurement

CRP was measured in serum in all four hospitals. The laboratories did not report an exact CRP less than 0.6 mg/L at Oslo University Hospital and 3.0 mg/L at Vestre Viken Hospital Trust.

### 2.4. Statistical Analysis

CRP values were not normally distributed as indicated using the Shapiro–Wilk test (W = 0.126, *p* < 0.001) and are reported as medians with ranges. Analyses were performed unadjusted and adjusted for GA, BW, and Apgar scores using multiple linear regression. Associations between CRP and other continuous variables were examined using Spearman’s rank correlation. Group comparisons were performed using Student’s *t*-test or the Mann–Whitney *U* test, as appropriate. Our primary outcome was CRP at 1 h, 12 h, and 36 h, with a main focus on infants who did not fulfill sepsis diagnostic criteria at the two hospitals with CRP screening, with subgroup analyses performed for (1) infants treated with antibiotics for suspected sepsis but where suspicion was not confirmed clinically or biochemically and (2) infants with the ten highest CRP values at 1 h and 36 h. The proportion of CRPs below or above the thresholds of 5, 10, 20, and 30 mg/L was calculated for different infant subgroups. Our secondary outcome was CRP in infants exposed to stress during and at birth, as evidenced by maternal and delivery characteristics, and low Apgar scores. Data on infants diagnosed with sepsis were used for reference. Limited data from the two non-screening hospitals were used for comparison. A *p*-value < 0.05 was considered significant. SPSS v29 (SPSS Inc., 2016, Armonk, NY, USA) was used for all analyses. Graphical figures were made in SPSS v29 and STATA 17 (StataCorp, College Station, TX, USA).

## 3. Results

### 3.1. Demographic Data

Characteristics of the 3154 included infants are presented in Table 1. The majority (2647 [83.9%]) were born vaginally (671 [21.3%] with vacuum and/or forceps).

### 3.2. CRP Related to Clinical Characteristics

#### 3.2.1. CRP in Infants Who Did Not Fulfill Diagnostic Criteria for Sepsis

**Figure 1:**
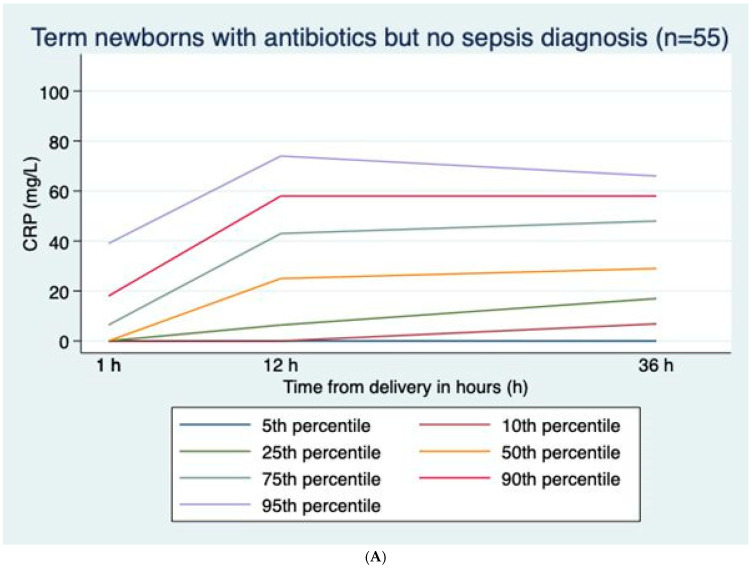

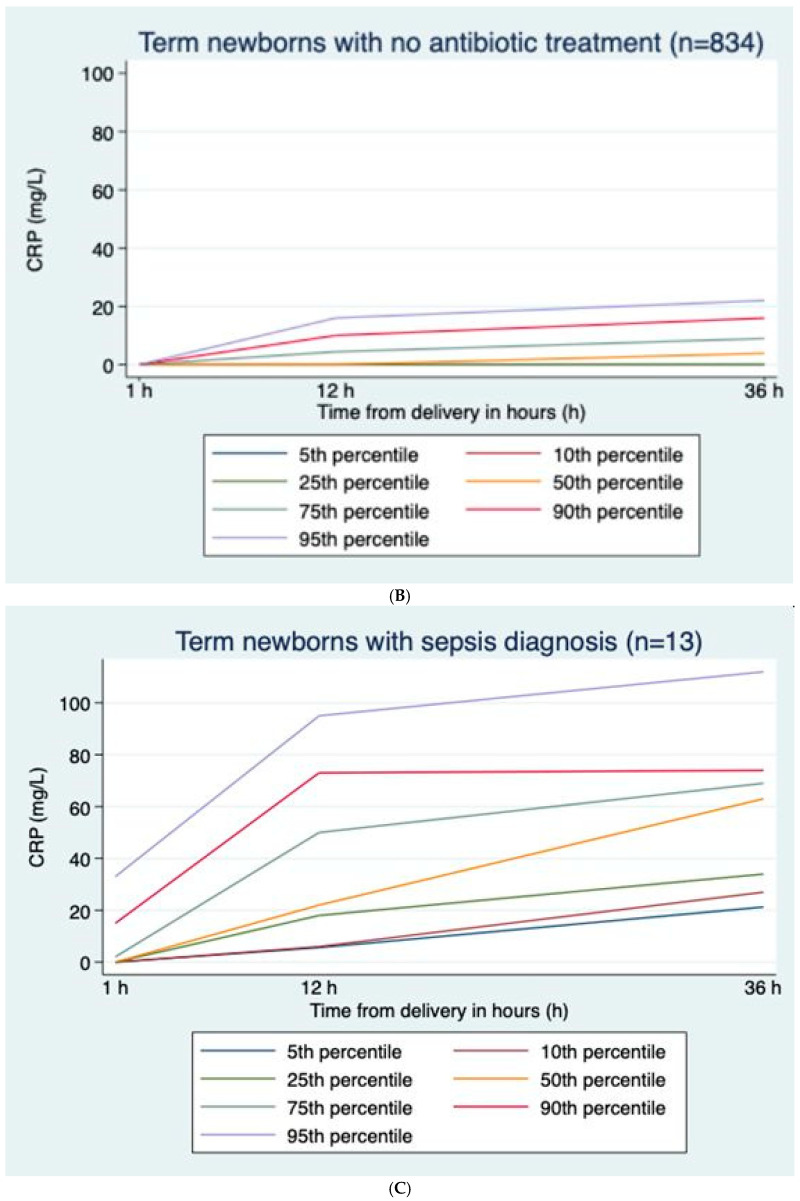
(**A**) C-reactive protein (CRP) curves with percentiles for term newborns with prolonged rupture of membranes and antibiotic treatment but no sepsis diagnosis; n = 55. (**B**) C-reactive protein (CRP) curves with percentiles for term newborns with prolonged rupture of membranes and no antibiotic treatment; n = 834. (**C**) C-reactive protein (CRP) curves with percentiles for term newborns with prolonged rupture of membranes diagnosed with sepsis; n = 13.

CRP values were positively skewed at all time points; median (range) values were 0 (0–62), 0 (0–82), and 4 (0–92) mg/L at 1, 12, and 36 h, respectively (Figure 1A), with corresponding percentages with CRP > 30 mg/L of 0.3%, 3.8%, and 4.8%, respectively. Excluding infants treated with antibiotics, median (range) values were 0 (0–18), 0 (0–59), and 4 (0–44) mg/L at 1, 12, and 36 h, respectively (Figure 1B), with corresponding percentages with CRP > 30 mg/L of 0%, 1.0%, and 1.9%, respectively (Table 2). In infants treated with antibiotics but not diagnosed with sepsis, median (range) values were 0 (0–62), 25 (0–82), and 29 (0–92) mg/L at 1, 12, and 36 h, respectively. CRP was higher in boys than girls at 12 h and 36 h, with median values of 5 (0–92) mg/L and 4 (0–112) mg/L, respectively (*p* < 0.001) at 36 h. CRP at 36 h was correlated to GA (r = 0.274, *p* < 0.001) and marginally to BW (r = 0.196, *p* < 0.001) and Apgar scores at 1 and 5 min (r = −0.11, *p* < 0.001; r = −0.091, *p* = 0.006). Both GA and BW were independent explanatory factors, but the effect of BW lessened when adjusting for GA.

In healthy infants and infants with sepsis, CRP rose gradually from ages 1 h to 12 h to 36 h, while in infants without sepsis treated with antibiotics, CRP rose from 1 h to 12 h and then gradually decreased towards 36 h (Figure 1A–C).

#### 3.2.2. CRP Values in Infants Not Receiving Antibiotics vs. Infants Receiving Antibiotics (Table 2 and Figure 1A,B)

Compared to infants never receiving antibiotics, CRP was markedly higher in infants treated with antibiotics, especially in infants fulfilling sepsis diagnostic criteria. Among infants who were not diagnosed with sepsis, the median CRP at 36 h was 29 mg/L in those receiving antibiotics and 0 mg/L in those who did not receive antibiotics (*p* < 0.001) (Figure 1A,B).

#### 3.2.3. CRP According to Maternal and Delivery Characteristics

There was no difference in infant CRP after vaginal delivery vs. C-section. Among infants delivered by C-section, CRP at 12 and 36 h was significantly higher when the C-section followed attempted vaginal delivery (median values 5 vs. 0 mg/L at 36 h, *p* = 0.021). Looking at vaginal deliveries without (n = 516) or with instruments (vacuum and/or forceps; n = 202), there was no difference in infant CRP at 1 h, but values at 12 and 36 h were significantly higher after instrumental deliveries, with 3 vs. 0 mg/L at 12 h and 6 vs. 4 mg/L at 36 h (*p*-values < 0.001). There was no difference in CRP between those with and without meconium-stained amniotic fluid and no correlation between CRP and PROM duration. There was no difference in CRP at any time point in the healthy infants of mothers with either proven group B streptococcus (GBS) (n = 55) or treated with peripartum antibiotics (n = 202). However, there was an increased risk for the infant to receive antibiotics but not for obtaining a sepsis diagnosis if the mother was treated with peripartum antibiotics (*p* = 0.001). Among infants treated with antibiotics, the CRP at 1 h was higher if the mother received peripartum antibiotics (*p* = 0.02). This finding was consistent across all hospitals.

#### 3.2.4. Highest CRP Values at Different Time Points

**Figure 2:**
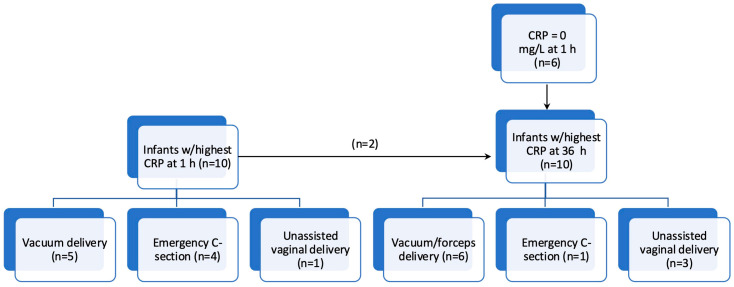
Flowchart showing the ten infants with the highest C-reactive protein (CRP) values at 1 h and 36 h, respectively, with delivery mode.

In the ten infants with the highest CRP at 1 h (15–62 mg/L), five (50%) were subjected to vacuum extraction, four (40%) were delivered by emergency C-section, and one (10%) was born vaginally without the use of instruments (Figure 2). None of the mothers were GBS-positive, but six (60%) were treated with peripartum antibiotics. All 10 (100%) infants were admitted to the NICU and received antibiotics with a median (range) treatment duration of 5 (3–6) days. Only one (10%) infant was diagnosed with sepsis, and none had a positive blood culture. None of these 10 infants received respiratory support.

Among the ten infants with the highest CRP at 36 h (63–112 mg/L), of whom only two were among the top ten at 1 h, six (60%) had a CRP at 1 h of 0 mg/L. Six (60%) were subjected to vacuum or forceps, one (10%) was delivered by emergency C-section, and three (30%) were born vaginally without the use of instruments (Figure 2). None of the mothers were GBS-positive and three (30%) received peripartum antibiotics. All infants were admitted to the NICU and received antibiotics with a median (range) treatment duration of 6 (3–20) days, where seven (70%) were diagnosed with sepsis and one (the infant treated for 20 days) had a positive blood culture (GBS). Two infants received respiratory support during NICU admission.

## 4. Discussion

In this study, we aimed to retrospectively examine CRP as a marker of inflammation in term infants born after PROM but not diagnosed with sepsis and to assess the correlation between CRP and different background variables and clinical characteristics. Although median CRP was low among term infants without sepsis born after PROM, we found CRP values above 60, 80, and 90 mg/L at 1, 12, and 36 h, respectively. Our results are consistent with those of Vasilescu et al. [14], who showed that PROM was a risk factor for EOS but that standard laboratory tests including CRP had low diagnostic specificity.

Contrary to Mjelle et al. [8], where only 1.1% of infants had CRP ≥ 30 mg/L, we found CRP > 30 mg/L in 4.8% of infants without sepsis at 36 h. However, only 1.6% had a CRP > 30 mg/L at 36 h when we excluded newborns treated with antibiotics. When establishing CRP reference values, it is not straightforward to exclude infants treated with antibiotics, as there is a circular reasoning fallacy present: an elevated CRP will in itself increase the risk of receiving antibiotic treatment, independent of clinical findings, and CRP was part of the sepsis diagnostic criteria in our study setting. Furthermore, we do not know whether infants with elevated CRP developed systemic infection at a later stage. Still, we speculate that a value above the Norwegian CRP diagnostic cutoff of 30 mg/L might not be appropriate in infants born after PROM. On the other hand, few infants with a sepsis diagnosis and none with culture-proven sepsis had a CRP above 30 mg/L at 1 or 12 h. CRP in infants diagnosed with sepsis was especially high at 36 h. Potential cutoff limits for “no sepsis” were <5 mg/L at 12 h and <20 mg/L at 36 h (Table 2). However, as the study was not designed to establish such cutoffs, these results should be considered with caution.

We have no definite explanation for the relationship between CRP and GA and BW within a cohort born at term. However, this finding is consistent with both earlier [8,9,15,16] and more recent [17] studies. One of the proposed mechanisms is increasing enzyme activity in the maturing liver and elevated placental cellular stress with increasing GA at and beyond term [18], with the same gradual increase described for umbilical cord blood lactate [19]. The effect of GA could not be explained by BW—on the contrary, although both variables were shown to be factors explaining CRP in linear regression analyses, we found that GA partly explained the effect of BW.

Our finding that CRP was higher in boys than in girls, also after adjusting for BW, contrasts with earlier studies in newborns [9,16] but is in agreement with Mjelle et al. [8] and reports from adults [20,21]. 

There was no difference in CRP between infants born vaginally vs. by C-section, but values were explicitly higher in cases where C-section followed attempted vaginal delivery. The lack of difference in CRP after vaginal delivery and C-section was expected, as almost all were emergency C-sections [6]. CRP also tended to be higher after instrumental than unassisted vaginal delivery, corresponding with earlier studies [22]. It has been found that the effect of instrumental delivery disappears after adjusting for the duration of labor [8,23]. We did not find that CRP was influenced by meconium staining or PROM duration. However, all infants were born after PROM ≥ 24 h, and it is possible that CRP reaches a maximum after a certain PROM length.

We found that infants of mothers treated with peripartum antibiotics but not of GBS-positive mothers were more prone to receiving antibiotics without fulfilling sepsis diagnostic criteria. There was also a slight increase in infant CRP at 1 h but not at 12 h and 36 h in these infants. We speculate that these infants had symptoms and increased CRP at 1 h due to maternal stress, prompting clinicians to initiate antibiotics despite guidelines stating EOS to be less likely in the event of peripartum antibiotics being administered.

Isolating the ten infants with the highest CRP values at 1 h and 36 h, we found that most cases were either instrumental vaginal deliveries or C-sections. All top ten infants at either 1 h or 36 h were admitted to the NICU and treated with antibiotics, but while 70% of the top infants at 36 h were diagnosed with sepsis, only 10% at 1 h fulfilled sepsis diagnostic criteria.

Limitations of the study were that we did not record maternal chorioamnionitis or predisposing factors for EOS and systemic inflammation other than PROM. Newer research hypothesizes a relationship between GBS colonization, maternal vaginal dysbiosis, and elevated markers of systemic inflammation [24,25]. As such, infant inflammatory markers, including CRP, should potentially be interpreted by more comprehensively considering maternal factors that we unfortunately did not account for in our study. We did not record maternal fever or the duration of labor, but we did have the exact number of hours of rupture of membranes. Prolonged labor is associated with a slight increase in CRP and may be related to PROM duration. Contrary to [10], we did not make comparisons with infants *without* PROM, and we did not include other biomarkers such as procalcitonin, white blood cell count, neutrophil count, and platelets. Another important limitation is that we could not rule out subclinical infection in infants classified as “healthy”, as we have no follow-up data beyond the infants’ first hospital admission. A newborn early warning score [26] performed at 2, 12, and 24 h after birth and at clinical indication was implemented in 2021 in Vestre Viken Hospital Trust and in 2023 at Oslo University Hospital. Such recent changes in clinical practice might limit the clinical applicability of this study’s results, as the newborn early warning score is based on clinical evaluation only and does not include biochemical tests.

Strengths of the study include the large number of infants and serial measurements of CRP at fixed intervals. However, more research on the potential role of CRP in EOS diagnostics is needed. The development of methods for salivary CRP measurements [27] might facilitate such research.

## 5. Conclusions

In term infants born after prolonged rupture of membranes but not diagnosed with early-onset neonatal sepsis, median CRP was low but with outliers above 60, 80, and 90 mg/L after 1, 12, and 36 h. These results differ from previous studies showing that CRP > 30 mg/L is uncommon in healthy term neonates and suggest that infants born after PROM may warrant particular attention beyond their increased risk of early-onset sepsis. Research is needed on the long-term outcomes of infants with inflammation, as evidenced by an elevated CRP, but not sepsis, after PROM.

## Figures and Tables

**Table 1:** Characteristics of deliveries and neonatal and maternal factors.

Delivery	Hospitals Without CRP Screening * (n = 2241)	Hospital with CRP Screening (n = 921)
**Mode of delivery, n (%)**		
Vaginal	1871 (83.5)	775 (84.1)
Emergency cesarean section	364 (16.2)	145 (15.7)
Elective cesarean section	6 (0.3)	1 (0.1)
**Use of instruments during vaginal delivery, n (%)**		
Vacuum extraction	422 (18.8)	196 (21.3)
Applied forceps	15 (0.7)	9 (1.0)
Vacuum and forceps	8 (0.4)	21 (2.3)
Missing	166 (7.4)	137 (14.9)
**Meconium characteristics, n (%)**		
Unremarkable	1727 (77.1)	674 (73.2)
Meconium stained	332 (14.8)	136 (14.8)
Bloodstained	64 (2.9)	50 (5.4)
Foul-smelling	1 (0)	1 (0.1)
Missing	115 (5.1)	59 (6.4)
**Neonatal characteristics**		
Female, n (%)	1125 (50.2)	433 (47)
Birth weight, g, mean (SD) (range)	3487 (458) (2088–5236)	3438 (444) (2110–5160)
Gestational age, weeks, mean (SD) (range)	39.5 (1.3) (37–45)	39.4 (1.2) (37–42)
Treated with antibiotics, n (%)	79 (3.5)	68 (7.4)
Early-onset sepsis, n (%)	25 (1.1)	13 (1.4)
Blood culture positive sepsis, n (%)	0 (0)	2 (0.2)
1 min Apgar, mean (SD)	8.68 (1.3)	8.69 (1.1)
5 min Apgar, mean (SD)	9.5 (0.9)	9.51 (0.8)
10 min Apgar, mean (SD)	9.82 (0.62)	9.85 (0.54)
CRP 1 h, median (range)	0 (0–48)	0 (0–62)
CRP 12 h, median (range)	4.4 (0–110)	0 (0–85)
CRP 36 h, median (range)	9.9 (0–171)	4.1 (0–112)
**Maternal characteristics**		
Peripartum antibiotic treatment, n (%)	707 (31.5)	243 (26.4)
GBS positive, n (%)	89 (4.0)	55 (6.0)

* At non-screening hospitals, CRP was measured only in infants tested on clinical indication, not as routine screening. CRP = C-reactive protein; GBS = group B streptococcus; SD = standard deviation.

**Table 2:** Percentage of infants with different C-reactive protein (CRP) threshold values at different time points, based on infant categories.

Newborns not receiving antibiotics (n = 834)	**1 h**	**12 h**	**36 h**
CRP > 30 mg/L, %	0%	1.0%	1.9%
CRP < 5 mg/L, %	98.9%	76.6%	57.8%
CRP < 10 mg/L, %	99.8%	89.3%	77.3%
CRP < 20 mg/L, %	100%	96.6%	93.3%
Newborns treated with antibiotics but not diagnosed with sepsis (n = 55)			
CRP > 30 mg/L, %	5.7%	48.1%	49.1%
CRP < 5 mg/L, %	71.7%	20.4%	9.1%
CRP < 10 mg/L, %	79.2%	31.5%	14.5%
CRP < 20 mg/L, %	90.6%	44.4%	29.1%
Newborns with sepsis diagnosis (n = 13)			
CRP > 30 mg/L, %	8.3%	38.5%	84.6%
CRP < 5 mg/L, %	83.3%	0%	0%
CRP < 10 mg/L, %	83.3%	15.4%	0%
CRP < 20 mg/L, %	91.7%	46.2%	0%
Newborns with blood culture-positive sepsis (n = 2)			
CRP > 30 mg/L, %	0%	0%	100%
CRP < 5 mg/L, %	100%	0%	0%
CRP < 10 mg/L, %	100%	50%	0%
CRP < 20 mg/L, %	100%	100%	0%

## Data Availability

The raw data supporting the conclusions of this article will be made available by the authors upon reasonable request.

## Abbreviations

The following abbreviations are used in this manuscript:

CRP	C-reactive protein
EOS	Early-onset neonatal sepsis
PROM	Prolonged rupture of membranes
GA	Gestational age
BW	Birth weight
GBS	Group B streptococcus
NICU	Neonatal intensive care unit
